# Changes in the medial prefrontal cortex metabolites after 6 months of medication therapy for patients with bipolar disorder: A ^1^H‐MRS study

**DOI:** 10.1111/cns.70048

**Published:** 2024-09-19

**Authors:** Haijin Li, Ju Gao, Huihui Song, Xuna Yang, Cai Li, Yue Zhang, Jiahui Wang, Yitong Liu, Dong Wang, Hong Li

**Affiliations:** ^1^ Department of Psychiatry The First Affiliated Hospital of Zhengzhou University Zhengzhou China; ^2^ Department of Geriatric Psychiatry, Suzhou Mental Health Center, Suzhou Guangji Hospital The Affiliated Guangji Hospital of Soochow University Suzhou China; ^3^ Department of Pharmacy The First Affiliated Hospital of Zhengzhou University Zhengzhou China

**Keywords:** bipolar disorder, depression, euthymia, glutamine, mania, medial prefrontal cortex, N‐acetyl aspartate, proton magnetic resonance spectroscopy

## Abstract

**Aims:**

The study aimed to assess brain metabolite differences in the medial prefrontal cortex (mPFC) between acute and euthymic episodes of bipolar disorder (BD) with both mania and depression over a 6‐month medication treatment period.

**Methods:**

We utilized ^1^H‐MRS technology to assess the metabolite levels in 53 individuals with BD (32 in depressive phase, 21 in manic phase) and 34 healthy controls (HCs) at baseline. After 6 months of medication treatment, 40 subjects underwent a follow‐up scan in euthymic state. Metabolite levels, including N‐acetyl aspartate (NAA), glutamate (Glu), and Glutamine (Gln), were measured in the mPFC.

**Results:**

Patients experiencing depressive and manic episodes exhibited a notable reduction in NAA/Cr + PCr ratios at baseline compared to healthy controls (*p* = 0.004; *p* = 0.006) in baseline, compared with HCs. Over the 6‐month follow‐up period, the manic group displayed a significant decrease in Gln/Cr + PCr compared to the initial acute phase (*p* = 0.03). No significant alterations were found in depressed group between baseline and follow‐up.

**Conclusion:**

This study suggests that NAA/Cr + PCr ratios and Gln/Cr + PCr ratios in the mPFC may be associated with manic and depressive episodes, implicating that Gln and NAA might be useful biomarkers for distinguishing mood phases in BD and elucidating its mechanisms.

## Graphical abstract

This study suggests that NAA/Cr + PCr ratios and Gln/Cr + PCr ratios in the mPFC may be associated with manic and depressive episodes, implicating that Gln and NAA might be useful biomarkers for distinguishing mood phases in BD and elucidating its mechanisms. ns not significant. *p < 0.05.

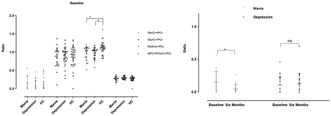

## 1 INTRODUCTION

Bipolar disorder (BD) is a heterogeneous and recurrent chronic psychiatric disorder, characterized by variable mood states that can be manic, depressive or mixed. Patients typically undergo periods of stability referred to as clinical remission in between these episodes. BD affects more than 1% of the global population and often results in cognitive impairment and a diminished quality of life.^1^, ^2^ Notably, individuals with BD commonly experience depressive episodes more frequently than manic ones, with many initially presenting with depressive symptoms, often leading to misdiagnosis as unipolar depression.^3^, ^4^, ^5^ This misclassification can have detrimental consequences for BD patients' prognosis.^4^ Consequently, there is a critical need to explore the underlying biological mechanisms of mood disorders to enhance accurate diagnosis and differential diagnosis. Despite numerous studies delving into the neurobiology of BD, our comprehension of this illness remains notably constrained, preventing definitive categorization or conceptualization based on etiology or mechanism due to insufficient knowledge.

The mPFC network plays a crucial role in the modulation of mood, cognitive processes, behavioral responses, and self‐referential processing.^6^, ^7^ Previous investigations found that mPFC in patients with BD had a decrease in volume, neuronal size/density and levels of metabolites.^8^, ^9^ A multimodal meta‐analysis of patients with BD displayed decreased voxel‐based morphometrics and increased activity in mPFC,^10^ which was consistent with a structural meta‐analysis of BD.^11^ Moreover, individuals who have smaller volumes of mPFC were found to exhibit riskier behavior during negative emotional states.^12^, ^13^ Recently, the other two multimodal meta‐analyses found aberrant function and structure within mPFC in patients with substance use disorder and obsessive‐compulsive disorder, which suggest that the mPFC might be associated with shared risk for psychiatric disorders.^14^, ^15^ These collective findings strongly indicated a potential association between the mPFC and emotional dysregulation as well as cognitive impairment in individuals with BD.

Proton magnetic resonance spectroscopy (^1^H‐MRS) is a professional non‐invasive imaging tool to quantify the metabolites in human brain.^16^, ^17^, ^18^ Previous cross‐sectional ^1^H‐MRS studies reported abnormalities in NAA levels among individuals with different subtypes and BD states. For example, decreased NAA or NAA/Cr levels have been observed within mPFC in patients with euthymic and depressive BD.^19^, ^20^, ^21^, ^22^ Atmaca M et al. observed decreased NAA/Cr ratios in first‐episode bipolar I patients.^23^ However, only one study has included both acute‐depressive and euthymic‐episode patients, which revealed reduced NAA/Cr in the basal ganglia.^4^ A consistent pattern of elevated glutamine/glutamate ratio or Glx (glutamate+glutamine) has been identified during euthymia or depression of BD.^18^, ^19^ GPC + PC levels have been shown to be low or normal in BD patients with depressive episode and high in BD patients in euthymia.^19^, ^24^ However, most prior MRS studies were performed in either euthymic or depressed bipolar patients or compared different populations in different mood statuses. A longitudinal investigation examining the neurometabolites in the mPFC among patients with BD during acute and euthymic episodes, encompassing both depressive and manic states, has not been undertaken to date.

The present investigation aims to examine longitudinal changes on mPFC neurometabolites in both BD patients experiencing depressive episode and manic episode following a six‐month medication regimen and investigate connection between metabolism changes and different clinical states. We focused on individuals during clinically depressive and manic episode in baseline, transitioning to clinical euthymia in month 6. In this study, we measured the levels of NAA, Glu, Gln and GPC + PC using 1H‐MRS and performed a semi‐quantitative analysis to quantify them. We also analyzed correlations between different mood states and the levels of metabolites in the mPFC. Based on the previous MRS researches of BD, we hypothesized that metabolite concentrations in the mPFC would manifest variations post‐treatment in individuals with acute‐episode BD, specifically concentrating on NAA and Gln. Additionally, we also hypothesized that fluctuations in mood states might align with changes in metabolite levels within the mPFC.

## 2 MATERIALS AND METHODS

This study was approved by the Ethics Committee of Suzhou GuangJi Hospital, Suzhou, China (No.2021–020). After a full explanation of the benefits and potential risks of the study. All participants provided written informed consent.

### 2.1 Participants

Fifty‐three BD subjects were followed up for 6 months of medication treatment. 32 BD patients with depressive episode and 21 BD patients with manic episode were included. All subjects were recruited and followed‐up at Suzhou GuangJi Hospital, Suzhou, China. To avoid the interference of aging and vascular disease, the participants aged from 18 to 55 years old.

All BD patients met the Diagnostic and Statistical Manual of Mental Disorders, fourth edition (DSM‐IV) criteria for BD assessed by the Structured Clinical Interview (SCID).^25^ Disease state was assessed with the 21item Hamilton Depression Rating Scale (HAMD)^26^ and the Young Mania Rating Scale (YMRS)^27^ at month 0 and 6. All enrolled BD patients were experiencing current acute episode of mania or depression at month 0.We recruited BD patients in an acute depressive episode scored ≥20 on HAMD and ≤5 on YMRS, and BD patients in an acute manic episode scored ≥18 on YMRS and ≤7 on HAMD. The BD patients who were remitted achieved scores of ≤7 on HAMD and ≤5 on YMRS. The exclusion criteria included: (1) other Axis I psychiatric disorders and symptoms, (2) a history of organic brain disease, brain trauma, or physical illness, (3) alcohol, drug or other psychoactive substance abuse or dependence, (4) a history of electroconvulsive therapy, (5) pregnant or breast‐feeding women, and (6) any contraindication to MRI scanning.

At follow‐up, all were clinically euthymic and none met DSM‐IV criteria for mania, hypomania or major depressive episode. All 53 BD subjects were scanned using ^1^H‐MRS at baseline, whereas 40 (22 from depression group and 18 from mania group) of the 53 completed the follow‐up scan after 6 months of treatment. All patients had been on medications on the days of MRI scan and during follow‐up.

Thirty‐four age‐ and gender‐matched HCs were enrolled. All HCs were screened according to the Structured Clinical Interview for DSM‐IV Nonpatient Edition (SCID‐NP) and had no psychiatric illness in self or in first‐degree relatives/personal or family history of mental illness. All participants were Chinese Han people and right‐handed.

### 2.2 Magnetic resonance data acquisition

**FIGURE 1:**
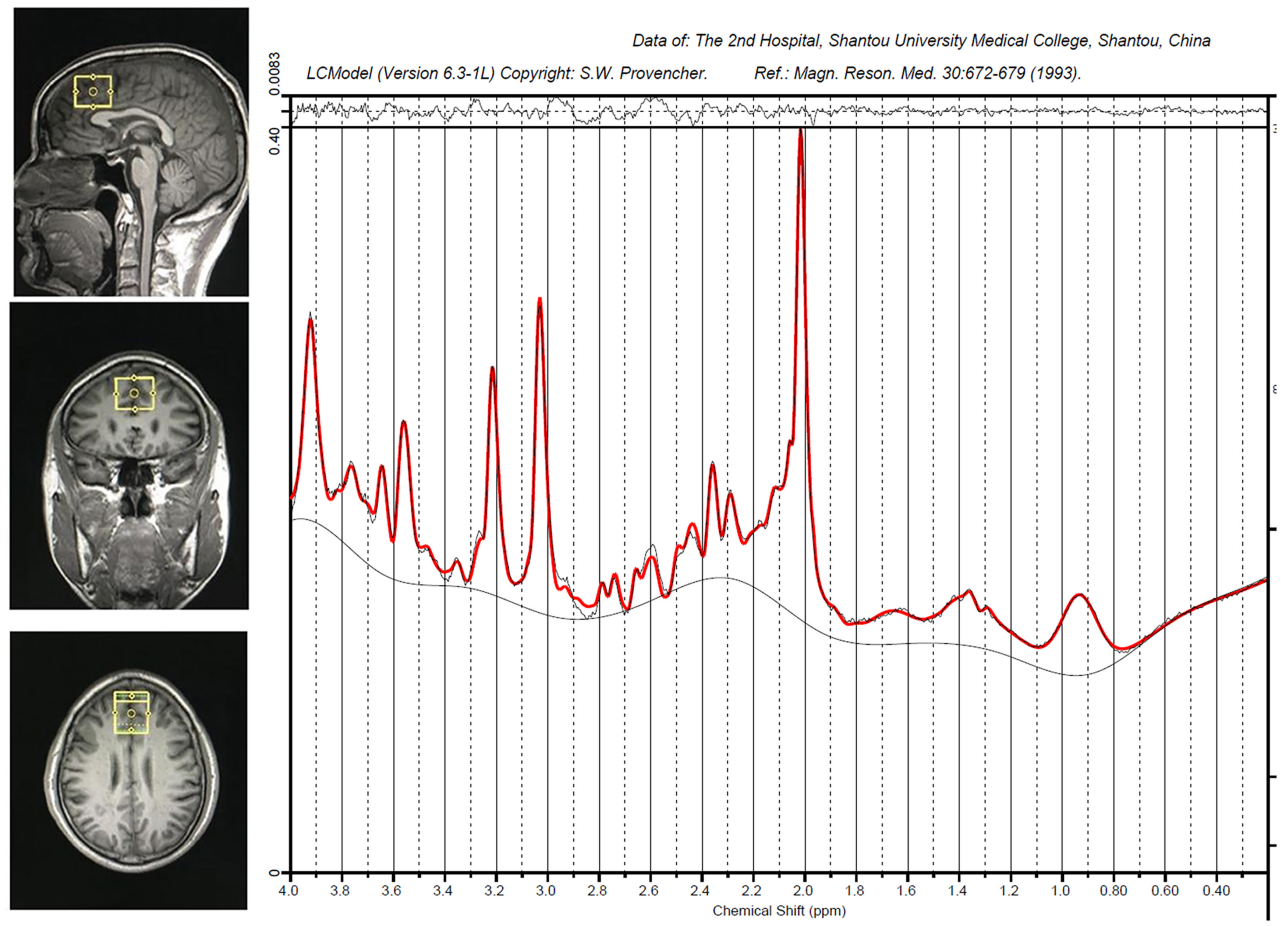
The location and sample spectrum of the voxel of interest in the medial prefrontal cortex (mPFC).

Structural MRI and MRS data were collected at Hunan Provincial People's Hospital in China utilizing a Siemens Magnetom Trio 3.0 T MR scanner by the same researcher. Three‐dimensional T1‐weighted images were obtained with specific parameters: repetition time of 2000 ms, echo time of 2.26 ms, field of view of 256 × 256 mm, flip angle of 8 degrees, matrix size of 256 × 256, 176 slices, and 1 mm slice thickness. The VOI measuring 20 × 20 × 20 mm was positioned in the gray matter of the mPFC, anterior to the genu corpus callosum, aligned with the anterior‐to‐posterior commissure (AC‐PC) line, using coronal, sagittal, and transverse images (Figure 1). The 1H‐MRS was performed using a PRESS sequence with water suppression achieved through a CHESS sequence (PRESS parameters: repetition time of 1500 ms, echo time of 30 ms, voxel size of 30 × 25 × 30 mm, 256 scans, spectral bandwidth of 1200 Hz, and 1024 data points).

### 2.3 MRS data analysis

The ^1^H‐MRS spectra underwent analysis using the LCModel version 6.3‐1B at the Second Affiliated Hospital of Shantou University Medical College in Guangdong, China. The raw spectra were fitted to key resonances of various common metabolites, such as NAA, NAAG, Glu, Gln, Cr, PCr, GPC, PCh, and mI, as shown in Figure 1. The unsuppressed water signal in mPFC served as an internal reference for the precise quantification of metabolites. Quality assessment of each spectrum included evaluation of the signal‐to‐noise ratio (S/N) and full width at half maximum (FWHM) to ensure suitability for accurate peak fitting of the targeted metabolites. Only metabolite spectra meeting the following criteria were included in the analysis: (1) FWHM ≤0.1 ppm; (2) S/N ratio ≥20; (3) Cramer–Rao lower bound (CRLB) <20%, resulting in the exclusion of 3 Glu values, and 9 NAA values from the statistical analyses. We performed a semi‐quantitative analysis to quantify the levels of NAA, GPC + PC, Gln and Glu of the mPFC in BD subjects. Metabolite levels were reported as ratios to Cr + PCr here. Cr + PCr was utilized as a standard for other metabolite peaks assuming its concentration remained relatively constant. The general practice of normalization by Cr + PCr eliminated the across‐subject variability, which originated from technical factors, such as coil loading.

Freesurfer version 6.0 was used for the segmentation of T1‐weighted MRI scans, dividing them into gray matter (GM), white matter (WM), and cerebrospinal fluid (CSF). The GM, WM, and CSF fractions for individuals with BD and those in the control group were standardized through the utilization of a tailored Matlab script (The Mathworks, Inc.), as detailed in Table 1.

**TABLE 1:** Demographic information, MRS data of participants.

Variable	BD subjects	Controls	*p*	T/*X* ^2^
	(*N* = 53)	(*N* = 34)		
Age(years old)	25.32 ± 4.84	26.32 ± 3.45	0.30	−1.05
Education(years)	12.32 ± 2.34	12.85 ± 1.79	0.26	−1.13
BMI	22.74 ± 1.76	23.39 ± 2.15	0.13	−1.53
CSF fraction	0.15 ± 0.04	0.15 ± 0.03	0.95	−0.06
Gray matter fraction	0.49 ± 0.04	0.49 ± 0.03	0.96	0.046
White matter fraction	0.35 ± 0.02	0.35 ± 0.02	0.97	0.043

*Note*: Date is presented as means±standard deviation.

Abbreviation: BMI body mass index, CSF cerebrospinal fluid.

### 2.4 Statistical analysis

All statistical analyses were conducted utilizing SPSS 26 software (SPSS Inc). The normality of data was assessed through the Kolmogorov–Smirnov test. Demographic variables were scrutinized using either the one‐way Chi‐squared test, analysis‐of‐variance (ANOVA) or Student's *t*‐test, as appropriate. Group differences in metabolite ratios were investigated using one‐way analysis of covariance (ANCOVA). Subsequently, in cases where significant differences emerged from the aforementioned comparisons, post hoc tests with Bonferroni correction were conducted to identify the specific group(s) responsible for the disparities. The paired *t*‐test was used to compare metabolite ratios and YMRS/HAMD scores between baseline and follow‐up assessments. Furthermore, partial correlation analyses were performed to evaluate the relationship between changes in metabolite ratios and alterations in HAMD/YMRS scores and the number of previous episodes, the duration of the illness.

## 3 RESULTS

### 3.1 Demographic and clinical characteristics at baseline

No notable variations were detected in age, educational attainment, BMI, CSF fraction, gray matter fraction, or white matter fraction across the cohorts, as delineated in Table 1. There was no difference in gender between BD patients undergoing depressive and manic episodes (*p* = 0.62). During the follow‐up period, medications taken by BD patients are shown in Table 2. In the cohort of BD patients in depressive episode, all individuals were prescribed a variety of medications including lithium (*n* = 6), valproate (*n* = 17), aripiprazole (*n* = 4), quetiapine (*n* = 18), olanzapine (*n* = 11), and antidepressants (*n* = 18). Similarly, in the group of BD patients with manic episode, all participants were also receiving treatment with lithium (*n* = 7), valproate (*n* = 13), aripiprazole (*n* = 5), quetiapine (*n* = 10), olanzapine (*n* = 6), and notably, no antidepressants.

**TABLE 2:** Clinical and demographic information of bipolar patients.

Variable	Depression	Mania	*p*	T/*X* ^2^
	(*N* = 32)	(*N* = 21)		
Gender (female/male)	19/13	11/10	0.62	0.25
Lithium (yes/no)	6/26	7/14		
Valproate (yes/no)	17/15	13/8		
Aripiprazole (yes/no)	4/28	5/16		
Quetiapine (yes/no)	18/14	10/11		
Olanzapine (yes/no)	11/21	6/15		
Antidepressants (yes/no)	18/14	0/21		
YMRS (M ± SD)	2.31 ± 1.06	20.19 ± 2.69	< 0.001	
HAMD (M ± SD)	27.78 ± 4.11	3.29 ± 1.15	< 0.001	
Illness duration (months) (M ± SD)	49.06 ± 21.05	51.52 ± 24.62	0.70	−0.39
Number of previous episodes (M ± SD)	3.47 ± 1.14	3.67 ± 1.24	0.55	0.55

Abbreviation: HAMD, The Hamilton Depression Rating Scale; M, Mean; SD, Standard deviation; YMRS, The Young Mania Rating Scale.

The averages of YMRS/HAMD points were presented with standard deviation. Specifically for BD individuals during depressive episode, the HAMD score was 27.78 ± 4.11 and the YMRS score was 2.31 ± 1.06. Among patients experiencing manic episodes in BD, the YMRS and HAMD scores were 20.19 ± 2.69 and 3.29 ± 1.15, respectively. The average duration of illness was 49.06 ± 21.05 months for BD subjects in the depressive state and 51.52 ± 24.62 months for BD subjects in the manic state. Additionally, the average number of episodes was 3.47 ± 1.14 in patients with depressive episode and 3.67 ± 1.24 in patients with manic episode. There was no significant difference in illness duration and number of previous episodes between BD patients in manic and depressive statuses, as shown in Table 2.

### 3.2 Quality of ^1^H‐MRS spectra

There were no substantial variations observed in gray matter (GM), white matter (WM), and cerebrospinal fluid (CSF) between individuals with BD and healthy controls (HC), as illustrated in Table 1. Furthermore, no noteworthy distinctions were identified in signal‐to‐noise ratio (SNR) and full width at half maximum (FWHM) across the three cohorts, as detailed in Table 3.

**TABLE 3:** Metabolite concentrations and FWHM and SNR comparations in BD patients and healthy controls.

Variable	M ± SD			ANCOVA^a^	D vs.M	D vs.HC	M vs.HC
	Depression	Mania	HC				
	(*N* = 32)	(*N* = 21)	(*N* = 34)				
Gln/Cr + PCr	0.10 ± 0.12	0.13 ± 0.15	0.10 ± 0.15	*F* = 0.41 (0.66)			
Glu/Cr + PCr	0.88 ± 023	0.84 ± 0.28	0.87 ± 0.28	*F* = 0.18 (0.84)			
NAA/Cr + PCr	0.96 ± 0.14	0.95 ± 0.18	1.10 ± 0.16	*F* = 7.33 (0.001)*	1	0.004*	0.006*
GPC + PCh/Cr + PCr	0.30 ± 0.04	0.28 ± 0.06	0.27 ± 0.06	*F* = 2.33 (0.10)			
Cr + PCr	4.86 ± 0.53	5.05 ± 0.90	5.04 ± 0.68	*F* = 0.74 (0.48)			
FWHM	0.05 ± 0.02	0.05 ± 0.02	0.06 ± 0.02	*F* = 0.11 (0.89)			
SNR	35.69 ± 12.13	33.00 ± 10.41	36.82 ± 6.37	*F* = 0.99 (0.37)			

*Note*: The three columns on the right‐hand side are results of post‐hoc tests, Bonferroni corrected.

Abbreviation: ANCOVA, analysis of covariance; Cr + PCr, creatine +phosphocreatine; D, Depression; Gln, glutamine; Glu, glutamate; GPC + PCh, glycerophosphocholine+ phosphocholine; M, Mania; M, Mean; NAA, N‐acetyl‐aspartate; SD, standard deviation.

^a^ the levels of metabolites were compared among the three groups using ANCOVA, with age, BMI, and education level as covariates.

*p < 0.05.

### 3.3 Comparison of baseline and 6‐month metabolite ratios during different groups

Compared with healthy controls, both BD patients in depressive and manic states had significant decreased NAA/PCr + Cr ratios (mean = 0.96 vs. 1.10, *p* = 0.004; mean = 0.95 vs. 1.10, *p* = 0.006). There was no difference among BD patients and control group in other metabolite ratios (details shown in Table 3). In 6‐month follow‐up, patients in euthymic status showed significant decline in Gln/Cr + PCr (t = 2.45, *p* = 0.03), compared with their acute‐manic episode, but no significant alterations were found in other metabolites levels (Table 4). Besides, we observed significant decreases of YMRS scores and HAMD scores in patients in euthymia, relative to their manic and depressive states separately. Patients in euthymic phase showed no differences in metabolites levels relative to their depressive phase (Table 4).

**TABLE 4:** Longitudinal changes in metabolites after 6‐month follow‐up period using 1H‐MRS.

	Baseline	Month 6	*t*	*p*
Mania				
Gln/Cr + PCr	0.14 ± 0.16	0.04 ± 0.07	2.45	0.03*
Glu/Cr + PCr	0.86 ± 0.24	0.92 ± 0.22	−0.82	0.42
NAA/Cr + PCr	1.02 ± 0.22	1.12 ± 0.10	−1.96	0.07
GPC + PCh/Cr + PCr	0.27 ± 0.06	0.29 ± 0.05	−1.36	0.19
Glu + Gln/Cr + PCr	1.00 ± 0.29	0.96 ± 0.24	0.48	0.63
Cr/PCr	4.95 ± 0.59	4.95 ± 0.44	−0.02	0.99
YMRS	19.94 ± 2.79	2.72 ± 0.83	31.22	<0.001***
Depression				
Gln/Cr + PCr	0.10 ± 0.13	0.13 ± 0.16	−0.53	0.60
Glu/Cr + PCr	0.83 ± 0.29	0.87 ± 0.22	−0.46	0.65
NAA/Cr + PCr	0.95 ± 0.26	1.02 ± 0.17	−1.03	0.32
GPC + PCh/Cr + PCr	0.29 ± 0.07	0.29 ± 0.04	−0.03	0.97
Glu + Gln/Cr + PCr	0.93 ± 0.05	0.99 ± 0.31	−0.55	0.59
Cr/PCr	5.01 ± 0.48	4.81 ± 0.55	1.48	0.15
HAMD	27.59 ± 4.06	4.55 ± 1.14	28.37	<0.001***

*Note*: Data are expressed as mean ± standard deviation.

*p < 0.05.

***p < 0.001.

### 3.4 Correlations between neurometabolite ratios and clinical data after follow‐up period

After a 6‐month follow‐up, there were no significant associations found between changes in YMRS or HAMD scores and variations in Glu/Cr + PCr (*r* = −0.03, *p* = 0.91; *r* = 0.34, *p* = 0.12), Gln/Cr + PCr (*r* = 0.19, *p* = 0.46; *r* = 0.34, *p* = 0.12), NAA/Cr + PCr (*r* = 0.10, *p* = 0.70; *r* = −0.07, *p* = 0.75), GPC + PCh/Cr + PCr (*r* = −0.09, *p* = 0.73; *r* = 0.20, *p* = 0.37) in BD patients in euthymia. We also did not find correlation between the number of previous episodes, the duration of the illness, and variations in metabolic profiles (all *p* values >0.05) (Table 5).

**TABLE 5:** Correlations between metabolite alterations with symptom changes and characteristics of the illnesses in BD patients.

Deviation	YMRS		HAMD		Illness duration(months)		Number of previous episodes	
	*r*	*p*	*r*	*p*	*r*	*p*	*r*	*p*
Glu/Cr + PCr	−0.03	0.91	−0.34	0.12	−0.22	0.39	−0.11	0.67
Gln/Cr + PCr	0.19	0.46	−0.17	0.45	0.28	0.26	0.11	0.66
NAA/Cr + PCr	0.10	0.70	−0.07	0.75	0.38	0.12	−0.23	0.35
GPC + PCh/Cr + PCr	−0.09	0.73	0.20	0.37	−0.024	0.92	−0.26	0.30

## 4 DISCUSSION

To our knowledge, this study represents the inaugural longitudinal investigation utilizing a 3.0 T multi‐voxel^1^ H‐MRS approach to assess variances in neurometabolites within the mPFC of individuals with remitted and acute‐episode BD manifesting mania and depression. In fact, most relevant studies that looked at the level of metabolites in the mPFC are cross‐sectional and rarely compared depressive, manic and euthymic status in same patients. To fill this gap, the primary objective of this longitudinal inquiry was to discern alterations in neuro‐metabolism among a sample of BD patients exhibiting depressive and manic episodes, who later achieved a euthymic state following 6 months of pharmacological treatment and intervention. Furthermore, this study sought to establish potential associations between MRS metrics in the mPFC region and the state of illness, encompassing depressive or manic episodes as well as euthymia.

A notable reduction in the NAA to Cr + PCr ratio was observed in both BD individuals with depressive and manic episodes compared to healthy controls (HCs) at baseline, with no significant variance noted after follow‐up. The decreased NAA levels across all mood states in BD may indicate an inherent susceptibility to the disorder. Furthermore, a consistent reduction in NAA levels was observed in individuals with BD, irrespective of their current mood state, suggesting that this decrease may manifest early in the course of the disorder and serve as a potential biochemical marker for acute episodes, implicating a close association with the pathophysiology of BD. This finding aligns with previous studies examining NAA levels in BD patients.^19^, ^21^, ^22^, ^24^, ^28^, ^29^, ^30^, ^31^, ^32^, ^33^, ^34^ For example, Li et al. found lower NAA levels in the mPFC of medication‐free individuals with bipolar depression compared to healthy controls.^19^ Reduced NAA levels were also observed in the mPFC and frontal lobe structures of euthymic BD patients.^21^, ^30^ Moreover, decreased NAA/Cr levels were noted in the left prefrontal white matter lobe in individuals with depressed BD, and in the bilateral white matter in patients with bipolar II depression.^22^, ^29^ The above studies indicated that reduced NAA might be associated with the pathogenesis of BD, but most of them only focus on one clinical state. Longitudinal studies revealed an increase in NAA levels in the mPFC and anterior cingulate cortex (ACC) of remitted BD adolescents, suggesting that NAA elevations may correspond to the resolution of manic symptoms,^28^, ^31^ indirectly supporting the hypothesis that decreased NAA ratios could indicate acute episodes of BD. However, it is essential to consider the potential impact of medications, particularly lithium, which has been proposed to exert neuroprotective effects or enhance neuronal function, potentially increasing or normalizing NAA levels.^24^, ^33^ Conversely, valproate administration in adults with BD has been associated with decreased NAA levels.^32^, ^34^

NAA is a vital marker in MRS used to assess neuronal function, viability, and quantity.^35^, ^36^ It is synthesized in neurons at high concentrations and is closely linked to mitochondrial energy production from Glu, indicating the functional status of neuronal mitochondria.^36^, ^37^ Reduced NAA levels may indicate compromised neuronal or mitochondrial function in BD, whereas increased NAA levels are associated with enhanced neuronal function and responsiveness to treatment.^38^ Neuroimaging studies have revealed a reduction in neurons and glia in BD, particularly in the prefrontal cortex white matter where decreased NAA levels correspond to alterations in neuroplasticity and synaptic plasticity in BD patients.^39^, ^40^, ^41^ Our results indirectly supported the above findings and suggested ongoing neuronal, synaptic, or mitochondrial dysfunction in the mPFC in individuals with BD despite clinical status, with NAA potentially playing a role in the onset and recovery of manic or depressive symptoms.

The subsequent discovery reveals that compared to their acute manic episode, individuals with BD during a state of euthymia exhibit a noteworthy reduction in Gln/Cr + PCr levels in the mPFC, while there was no significant variance in Gln level in the mPFC between BD patients with depressive episode at the baseline and the sixth month. Also no difference was found in the Gln/Cr + PCr level in mPFC among patients with manic and depressive episode and HCs in baseline. Previous cross‐sectional MRS investigations have found aberrant Gln/Cr + PCr level in BD. For example, A systematic review of MRS studies has indicated a consistent trend of decreased Glx levels in depression and increased levels in mania.^42^ Escalated glutamine/glutamate ratio or Glx levels have been documented in acute mania in the anterior cingulate cortex or left dorsolateral prefrontal cortex (DLPFC).^43^, ^44^ A meta‐analysis by Gigante et al. showed increased Glx levels in BD.^45^ Elevated Gln levels have also been identified in the euthymic phase of BD depression.^18^ These findings are inconsistent with our comparison in baseline. The discrepancies may stem from various factors such as heterogeneity of subjects, difference of region of interests, sample size and the effect of previous medications taken. Particularly, extended utilization of mood stabilizers such as lithium and valproate may lead to reductions in Glx levels due to multiple mechanisms.^3^, ^46^, ^47^

The glutamatergic system is known to play a crucial role in the pathophysiology of BD, as supported by previous studies^28^, ^45^, ^48^ and findings from our own research directly. Glutamate, initially released by neurons, undergoes conversion to glutamine in astrocytes, and is then transformed back to glutamate in neurons through the “glutamate‐glutamine cycle”.^49^ Research has shown that glutamine levels can act as indicators of glutamatergic neurotransmitter activity, reflecting the complex interactions between neurons and glial cells.^50^ Given the essential role of Glial cells in glutamate and glutamine maintenance/recycling, elevated glutamine may implicate glial dysfunction and impaired glial‐neuronal interactions, impacting glutamate processing. Discrepancies in Gln/Cr + PCr ratios have been observed between BD and Major Depressive Disorder (MDD), with deficits in glial cells noted in both conditions.^40^ Studies on proton magnetic resonance spectroscopy have suggested that depressive and manic episodes may involve opposing changes in the glutamine/glutamate ratio, indicating potential reductions in glutamate conversion to glutamine in depression and elevations during manic episodes.^42^ The present study revealed a tendency for Gln levels to decrease over time in the manic episode group, relative to their initial levels, which, although not indicative of a consistent elevation in manic episodes compared to depression and HCs, suggests a complex role of Gln metabolism in the pathophysiology of BD episodes. Further investigation is warranted to clarify Gln's potential as a marker or mediator in the differential diagnosis and pathomechanism of BD mood phases.

Unfortunately, this study did not identify a significant relationship between changes in metabolite ratios and alterations in YMRS/HAMD scores. Giselli's research team noted that structural brain modifications and cognitive impairments are not consistently observed in the early stages of BD, but may become more pronounced with increased chronicity and number of episodes.^51^ The absence of notable differences could be attributed to variations in the duration of illness and the number of episodes experienced by BD subjects. Likewise, André Ehrlich et al. detected no association between NAA or Glu in the ACC and HAMD score, or YMRS score in bipolar subject.^52^ Zhong similarly reported no significant link between NAA/Cr levels and HAMD scores in white matter areas such as the prefrontal lobe, anterior cingulate cortex, and hippocampus.^22^ This lack of correlation may due to the limited sample size, illness diversity or medication effects. Therefore, the findings of this research are considered preliminary, emphasizing the need for subsequent longitudinal studies involving a larger cohort, an extended follow‐up duration, and individuals with first‐episode or medication‐free bipolar disorder. Additionally, it is recommended to gather longitudinal data from healthy controls and obtain multimodal functional magnetic resonance imaging data of the medial prefrontal cortex to enhance the robustness of the conclusions.

## 5 LIMITATIONS

The study has some limitations that should be acknowledged. Firstly, the sample size was small, potentially impacting the statistical power of the analyses. Another limitation is the varied medications used during follow‐up, which could affect metabolite levels. Future research should aim to exclude medication effects for a clearer understanding of biochemical changes in BD patients. Besides, the previous episodes and medication use of BD subjects may bring effects to our comparisons. In future, further longitudinal investigations are needed to explore the relationship between metabolism and clinical states in untreated first‐episode BD patients with depressive and manic episode. It is also commended to monitoring longitudinal brain metabolic changes in healthy controls to strengthen researches.

## 6 CONCLUSION

Our research elucidates the involvement of mitochondrial energy metabolism in neuroplasticity and synaptic plasticity within BD. It identifies an anomaly associated with traits in individuals experiencing acute episodes of BD, presenting diminished NAA/Cr + PCr ratios in the mPFC. Fluctuations in NAA levels could indicate the presence of acute episodes or recovery from manic or depressive symptoms. The decreased tendency for Gln levels observed in patients in the manic state suggested a complex role of Gln metabolism in the pathophysiology of BD episodes. In conclusion, this study underscores the potential and importance of NAA and Gln irregularities in the mPFC as significant features in the progression and differential diagnosis of BD states.

## AUTHOR CONTRIBUTIONS

**Haijin Li:** Conceptualization, Methodology, Software, Writing‐original draft; **Ju Gao:** Methodology, Writing‐original draft; **Huihui Song:** Data collection and analysis; **Xuna Yang:** Data collection and investigation; **Cai Li:** Investigation, Resources; **Yue Zhang:** Data curation, Resources; **Jiahui Wang:** Investigation and Visualization; **Yitong Liu:** Investigation and data collection; **Dong Wang:** Conceptualization, Investigation, Methodology, Supervision, Writing‐review and editing; **Hong Li:** Data curation, Resources, Visualization, Supervision. All authors read and approved the final manuscript.

## FUNDING INFORMATION

This study was supported by the Jiangsu Natural Science Foundation of China (BK20211081), Henan Health Commission of China (HNSWJW‐2022009), Henan Scientific and Technological Development Program of China (No. 232102311051), Suzhou Key Science and Technology Project of China (SS202071), Suzhou Key clinical medical disciplines project of China (SZXK202116), Suzhou Key clinical treatment project of China (LCZX202327), Suzhou clinical Medical Center for mood disorders of China (Szlcyxzx202109), National Natural Science Foundation of China (81801325) and Henan Young and Middle‐aged Health Science and Technology Innovation Talent Training Program of China (YXKC2020035).

## CONFLICT OF INTEREST STATEMENT

The authors declare that the research dose not have any potential conflicts of interest about commercial or financial relationships.

## Data Availability

The data that support the findings of this study are available from the corresponding author upon reasonable request.
